# Partial Discharge Gas Generation Characteristics and Molecular Degradation Mechanisms of Cellulose Polymers in Eco-Friendly Insulating Oils

**DOI:** 10.3390/polym18121493

**Published:** 2026-06-14

**Authors:** Yiheng Zhou, Yixin He, Guangliang Liu, Xianglin Kong, Jiaming Yan, Wenyu Ye

**Affiliations:** 1 School of Electrical Engineering, China University of Mining and Technology, Xuzhou 221116, China; zhouyiheng55@163.com (Y.Z.); ts24230090p31@cumt.edu.cn (Y.H.); lgl1116lgl@163.com (G.L.); ts25230016a31@cumt.edu.cn (X.K.); yanjiaming888@163.com (J.Y.); 2 State Key Laboratory of Power Transmission Equipment Technology, School of Electrical Engineering, Chongqing University, Chongqing 400044, China

**Keywords:** cellulose insulation paper, bio-based insulating oil, oil-paper insulation, partial discharge, ReaxFF molecular dynamics, dissolved gases in oil

## Abstract

Two bio-based insulating oils (BHOs) with average carbon chain lengths of approximately 18 and 22 were investigated as short- and long-chain BHOs. By constructing an oil-paper composite insulation system, the generation law of characteristic gases in the two systems was studied by partial discharge experiments. Based on the ReaxFF reaction molecular dynamics simulation under electrothermal coupling stress, the cracking path, cracking rate, evolution of oxygen-containing small molecules, and generation path of characteristic gases of cellulose polymer were revealed. Both systems produced H_2_, CH_4_, C_2_H_2_, C_2_H_4_, C_2_H_6_, CO, and CO_2_, with CO_2_ dominant and C_2_H_6_ least abundant. The short-chain BHO generated markedly higher amounts of H_2_, CO, C_2_H_2_, and C_2_H_4_ than the long-chain BHO; after 15 min, its H_2_ and CO concentrations were about 3.4- and 2.1-times those in the long-chain system, respectively. ReaxFF simulations showed that cellulose degradation in the short-chain BHO followed stepwise chain scission and continuous decarbonylation, favoring CO and unsaturated gas precursors. In contrast, cellulose chains disappeared faster in the long-chain BHO, producing more oxygen-containing organic fragments and C_1_-C_5_ oxygenated molecules and a higher small-molecule conversion ratio. Characteristic gas pathway analysis revealed that all seven gases could be generated from cellulose pyrolysis intermediates, and different oil environments primarily influenced gas generation behavior by altering the evolution pathways of these intermediates. These findings, at the molecular scale, elucidate the impact of BHO environments on the degradation mechanism of cellulose polymers, providing a theoretical basis for the condition assessment and design of environmentally friendly oil-paper insulation systems.

## 1. Introduction

Oil-paper composite insulation is one of the most important insulation structures in oil-immersed power transformers, and its operational reliability is directly related to the safe service of electrical equipment and the stable operation of power grids [1]. Although conventional mineral insulating oils exhibit good dielectric and heat dissipation properties, they suffer from poor renewability, limited biodegradability, relatively high environmental risks, and comparatively low fire points [2,3]. In addition, mineral oils are derived from non-renewable petrochemical resources and tend to generate acidic oxidation products and moisture during long-term operation, which accelerates the aging of oil-paper insulation and reduces equipment reliability [4]. Therefore, the development of environmentally friendly and stable insulating oils has become an important direction for oil-paper insulation systems.

In recent years, natural ester and synthetic ester insulating oils have attracted increasing attention as alternatives to mineral oils [5,6]. Natural ester insulating oils possess good biodegradability and high fire points [7], but they still have several limitations, such as relatively high viscosity, poor low-temperature fluidity, and high dielectric loss [8]. Synthetic esters exhibit good thermal stability and oxidation resistance; however, their electrical properties still differ to some extent from those of mineral oils [9]. Unlike natural and synthetic esters, BHOs, mainly composed of isoalkanes, combine low viscosity, high volume resistivity, and excellent insulating properties [10,11,12]. They also show promising application potential in terms of breakdown voltage, flash point, and thermal stability [11,13]. Moreover, their low dielectric loss and favorable heat dissipation capability make them promising candidates for environmentally friendly oil-paper insulation systems [14].

In oil-paper composite insulation systems, insulating paper is mainly composed of cellulose. Cellulose is a typical natural polymer consisting of glucose units connected by β-1,4-glycosidic bonds. Its degree of polymerization, molecular chain integrity, and oxygen-containing functional groups directly affect the mechanical properties, dielectric performance, and service life of insulating paper [15,16]. During long-term operation, oil-paper insulation systems are subjected to multiple coupled stresses, including thermal stress, electric field stress, partial discharge, and moisture. Among them, partial discharge is one of the important factors inducing cellulose chain scission and insulation deterioration [17,18]. Recent studies on environmentally friendly insulating liquids have also shown that the evolution of partial discharge characteristics is closely related to cellulose aging and insulation state identification under electro-thermal aging conditions [19]. High-energy electrons, active radicals, and localized thermal effects generated during partial discharge can induce the cleavage of glycosidic bonds, C-O bonds, and C-H bonds in cellulose chains, further triggering dehydration, decarbonylation, decarboxylation, hydrogen transfer, and radical recombination reactions [20]. As cellulose chains continue to decompose, their degree of polymerization decreases, accompanied by the generation of H_2_, CO, CO_2_, and low-molecular-weight hydrocarbon gases, eventually leading to the degradation of the mechanical and dielectric properties of insulating paper.

Dissolved gas analysis is an important method for evaluating the condition of oil-paper insulation. Previous studies have shown that CO and CO_2_ are closely related to the decomposition of oxygen-containing structures in cellulose, while H_2_ and low-molecular-weight hydrocarbon gases are associated with dehydrogenation, chain scission, and radical evolution processes [14,15]. However, conventional gas chromatography can only provide macroscopic gas concentration variations and cannot directly reveal the bond-breaking positions, intermediate evolution pathways, or gas formation mechanisms of cellulose polymer chains under partial discharge. Therefore, macroscopic gas generation results alone are insufficient to explain the differences in cellulose degradation behavior under different insulating oil environments.

For BHOs, molecular structure and average carbon chain length can affect fundamental properties such as viscosity, thermal stability, and molecular mobility [11,13], thereby altering the diffusion, collision, and subsequent reactions of cellulose-derived intermediates in oil-paper composite systems. Although previous studies have investigated eco-friendly transformer insulating liquids, most of them have focused on natural esters, synthetic esters, mineral oils, or the general physicochemical and dielectric properties of alternative insulating liquids. The influence of BHO chain length on partial-discharge-induced cellulose degradation and characteristic gas generation remains insufficiently understood. In particular, the relationship among BHO chain length, cellulose chain scission, oxygen-containing intermediate evolution, and gas formation pathways has not been systematically revealed. Therefore, comparing short-chain and long-chain BHO environments can provide molecular-level insight into how the chain structure of insulating oil affects cellulose degradation and gas generation in oil-paper composite insulation systems.

Reactive molecular dynamics simulation provides an effective approach for investigating the degradation mechanisms of cellulose polymers [21]. ReaxFF-based reactive molecular dynamics can dynamically describe chemical bond breaking and formation, enabling the tracking of radicals, intermediates, and small-molecule gas formation pathways during polymer decomposition [22]. Compared with conventional molecular dynamics, ReaxFF can not only capture cellulose backbone cleavage and the transformation of oxygen-containing functional groups, but also further analyze the microscopic formation mechanisms of CO, CO_2_, H_2_, and low-molecular-weight hydrocarbon gases. Therefore, combining partial discharge experiments with ReaxFF reactive molecular dynamics simulations enables the degradation behavior of cellulose polymers in different BHO environments to be revealed from both macroscopic gas evolution and microscopic molecular reaction perspectives.

In this study, two BHOs with different average carbon chain lengths were investigated, which are denoted as short-chain BHO and long-chain BHO, with average carbon chain lengths of approximately 18 and 22, respectively. First, needle-plate defect partial discharge experiments were conducted to obtain the evolution characteristics of seven characteristic gases, namely H_2_, CH_4_, C_2_H_2_, C_2_H_4_, C_2_H_6_, CO, and CO_2_, in two oil-paper composite insulation systems. Subsequently, ReaxFF reactive molecular dynamics simulations were performed to analyze the degradation pathways, decomposition rate, evolution of oxygen-containing small molecules, and characteristic gas formation pathways of cellulose polymers under coupled electrical-thermal stress. This study aims to clarify the influence mechanisms of different BHO environments on cellulose decomposition behavior and partial-discharge-induced gas generation, providing molecular-level insights for condition assessment and insulation material optimization in environmentally friendly oil-paper insulation systems.

## 2. Experiment and Simulation Section

### 2.1. Sample Preparation and Pre-Treatment

Two BHOs supplied by Shanghai Dowell Company, Shanghai, China, were used in this study. Their main components were isoalkanes with different carbon chain lengths. The two insulating oils were denoted as short-chain BHO and long-chain BHO, with average carbon chain lengths of approximately 18 and 22, respectively. These two BHOs were selected as representative short-chain and long-chain bio-based hydrocarbon insulating oils. The short-chain BHO has a carbon number closer to the hydrocarbon components commonly found in conventional mineral insulating oils, whereas the long-chain BHO has a longer carbon chain structure and a higher flash point, representing a BHO with improved high-temperature safety characteristics. The basic physicochemical and electrical properties of the two BHOs are listed in Table 1. Before the experiment, the two insulating oils were separately injected into a vacuum oil filtration system and filtered at 60 °C to remove possible mechanical impurities. The solid insulating material used in the experiment was kraft insulating paper, with a thickness of 1 mm and a density of 1.2 g/cm^3^. Before the experiment, the insulating paper was cut into circular sheets with a diameter of 100 mm to meet the assembly requirements of the needle plate electrode partial discharge test. To reduce the influence of moisture and dissolved gases on the partial discharge behavior and gas generation characteristics of the oil-paper composite insulation system, the insulating oils and cellulose insulating paper were subjected to drying, degassing, and vacuum impregnation before the experiment [8,23]. First, the two BHOs were dried and degassed in a vacuum environment at 90 °C and 50 Pa for 48 h. At the same time, the insulating paper samples were dried in a vacuum oven at 90 °C and 50 Pa for 48 h to ensure that their moisture content was lower than 1%. Subsequently, the dried insulating paper samples were immersed in the short-chain BHO and the long-chain BHO, respectively, and were impregnated in a vacuum environment at 60 °C and 50 Pa for 24 h. This process allowed the insulating oil to fully penetrate the pores of the cellulose pressboard, thereby eliminating residual air gaps inside the insulating paper and obtaining stable oil-paper composite insulation samples.

### 2.2. Partial Discharge Test

**Figure 1:**
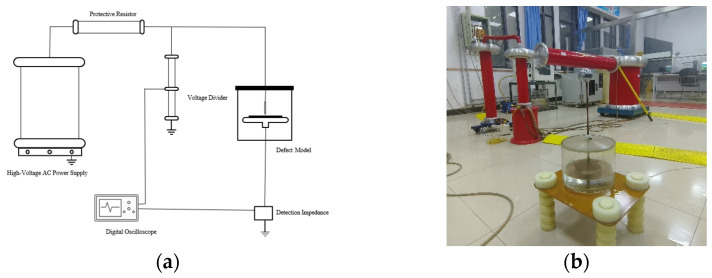
Schematic diagram and physical setup of the partial discharge (PD) detection platform. (**a**) Schematic diagram of the PD detection system. (**b**) Photograph of the PD experimental platform.

To simulate typical nonuniform electric field defects in transformer oil-paper insulation systems and investigate the degradation behavior of cellulose polymers under electric field stress, a partial discharge detection platform based on the pulse current method was established in this study. The schematic diagram and physical setup of the test platform are shown in Figure 1. The test system mainly consisted of an AC high voltage power supply, a test transformer, a protective resistor, a partial discharge detection impedance, and a digital oscilloscope. The output voltage range of the AC high voltage power supply was 0 to 50 kV, and the rated voltage of the test transformer was 50 kV, which provided a stable AC electric field for the oil-paper composite insulation samples. A 5 kΩ water resistor was used as the protective resistor to limit the impulse current during breakdown. The partial discharge signal was extracted using a laboratory made RLC detection impedance and recorded by a digital oscilloscope with a bandwidth of 400 MHz and a maximum sampling rate of 20 GS/s.

**Figure 2:**
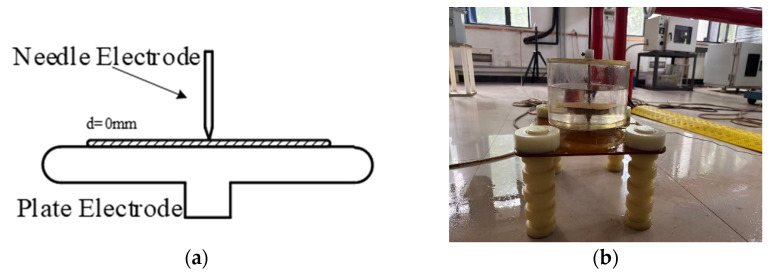
Needle-plate defect model. (**a**) Schematic diagram. (**b**) Physical model.

A needle plate defect model was used in the experiment, as shown in Figure 2. The needle tip was in direct contact with the upper surface of the insulating paper, and the gap distance was 0 mm. The plate electrode was placed beneath the insulating paper, and the distance between the needle tip and the plate electrode was equal to the thickness of the insulating paper, namely 1 mm. This structure could form a highly concentrated local electric field at the contact region between the needle tip and the paper surface, which was used to simulate a typical internal defect caused by a conductor tip burr in close contact with insulating paper in a transformer. The impregnated oil-paper composite samples were placed in a sealed oil cup to reduce the influence of external air and moisture on the partial discharge process and gas generation behavior.

Before the formal experiment, the partial discharge inception voltage, abbreviated as PDIV, was measured using the stepwise voltage rising method. Under a 50 Hz AC voltage, the voltage was increased from 0 kV at a rate of 0.5 kV/s. According to the IEC 60270 standard [24], when a continuous and stable partial discharge signal was observed on the oscilloscope, the corresponding voltage was recorded as the PDIV. Each oil-paper composite system was tested repeatedly, and the average value was used as the reference voltage for the subsequent constant voltage partial discharge test. The measured PDIV values of the short-chain and long-chain BHO oil-paper composite systems were 14.23 kV and 15.79 kV, respectively. The PDIV of the long-chain BHO system was approximately 10.96% higher than that of the short-chain BHO system, indicating that the long-chain BHO oil-paper system exhibited a slightly higher partial discharge inception resistance under the same needle-plate defect condition.

In the formal partial discharge experiment, the constant voltage method was adopted. The applied voltage was increased to 1.2-times the PDIV and then kept constant for 15 min to accelerate the development of partial discharge and the degradation process of cellulose polymers. The discharge duration was limited to 15 min because, under the severe needle–plate defect condition with a 0 mm oil-paper gap, the insulating paper generally broke down after approximately 15 min of continuous partial discharge. Therefore, 15 min was selected as the maximum duration for obtaining stable gas generation data before final breakdown. During partial discharge, high energy electrons, ion bombardment, active radicals, and local high temperature jointly induced glycosidic bond cleavage, dehydration, decarbonylation, and decarboxylation reactions in cellulose molecular chains, further generating H_2_, CO, CO_2_, and low molecular weight hydrocarbon gases. To track the gas generation behavior at different discharge stages, oil samples were collected at 5 min, 10 min, and 15 min of discharge and then sent to a gas chromatograph for dissolved gas analysis. To ensure the repeatability of the experimental results, each group of experiments was repeated four times, and the average value was used for analysis.

### 2.3. Dissolved Gas Analysis Based on Gas Chromatography

**Figure 3:**
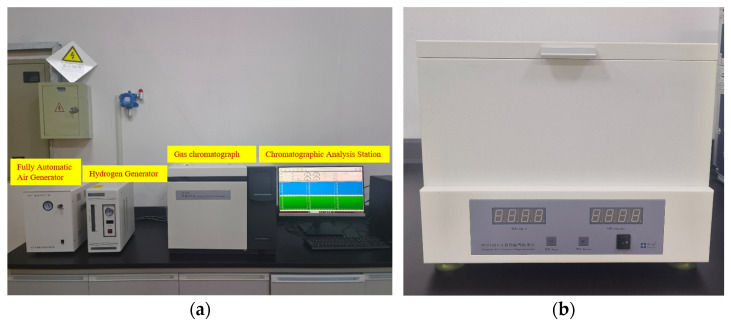
Equipment used for dissolved gas analysis: (**a**) gas chromatograph; (**b**) automatic degassing and vibration device.

To quantitatively analyze the small molecular gases generated from cellulose polymer degradation during partial discharge, gas chromatography was used to detect dissolved gases in oil. Gas chromatographic analysis separates and quantifies mixed gases based on the differences in the distribution behavior of different gas components between the stationary phase and the mobile phase. The detected gases included seven typical characteristic gases, namely H_2_, CH_4_, C_2_H_2_, C_2_H_4_, C_2_H_6_, CO, and CO_2_. Gas chromatographic detection was performed using a ZF-301 transformer oil gas chromatograph manufactured by Henan Zhongfen Instrument Co., Ltd., Shangqiu, China, as shown in Figure 3. The instrument was equipped with a thermal conductivity detector, abbreviated as TCD, for detecting H_2_. It was also equipped with a flame ionization detector, abbreviated as FID, for detecting CH_4_, C_2_H_2_, C_2_H_4_, C_2_H_6_, CO, and CO_2_. The hydrogen and air required for the FID were supplied by a GH-300 hydrogen generator and an SPB-3 fully automatic air source, respectively.

The degassing and injection procedures for oil samples were carried out according to the mechanical vibration dissolved equilibrium method specified in GB/T 17623-2017 [25]. First, 60 mL of oil sample was collected from the sealed oil cup after the experiment using a 100 mL glass syringe. After residual bubbles in the syringe were completely removed, the oil volume was adjusted to 40 mL, and the syringe was sealed with a rubber stopper. Subsequently, 10 mL of argon was injected into the oil sample syringe using a 10 mL glass syringe as the equilibrium gas. The syringe was then placed in an automatic degassing and vibration device at 50 °C for mechanical vibration, as shown in Figure 3b, so that the dissolved gases in the oil reached distribution equilibrium between the gas and liquid phases. After equilibrium was established, the gas in the upper part of the syringe was extracted using a 5 mL glass syringe, and the total volume of the extracted gas was recorded. Then, gas was taken using a 1 mL glass syringe and rapidly injected into the gas chromatograph for component detection. According to the extracted gas volume, gas liquid partition coefficient, and chromatographic peak area, the concentrations of each characteristic gas in the oil were calculated following the method provided in the appendix of GB/T 17623-2017.

### 2.4. Reaction Kinetics Simulation Based on ReaxFF

To reveal the decomposition behavior of cellulose polymers and the formation mechanisms of characteristic gases in different BHO environments at the molecular level, ReaxFF reactive molecular dynamics simulations were performed to investigate the degradation process of oil-paper composite systems under coupled electrical and thermal stress. Transformer insulating paper is mainly composed of cellulose. Cellulose is a linear natural polymer formed by β-D-pyran glucose units connected through β-1,4-glycosidic bonds, and its chemical formula can be expressed as (C_6_H_10_O_5_)_n_, where *n* represents the degree of polymerization. Previous studies by K. Mazeau showed that cellulose chains with different degrees of polymerization exhibit very small differences in molecular conformation and physicochemical properties [26]. Wang et al. further reported that when the degree of polymerization was equal to or higher than 10, the calculated mechanical and chemical properties became stable and conformed to the actual properties of cellulose [27]. Therefore, a cellulose chain with a degree of polymerization of 10 was selected as the representative polymer model in this study to characterize cellulose backbone cleavage, radical evolution, and characteristic gas formation.

**Figure 4:**
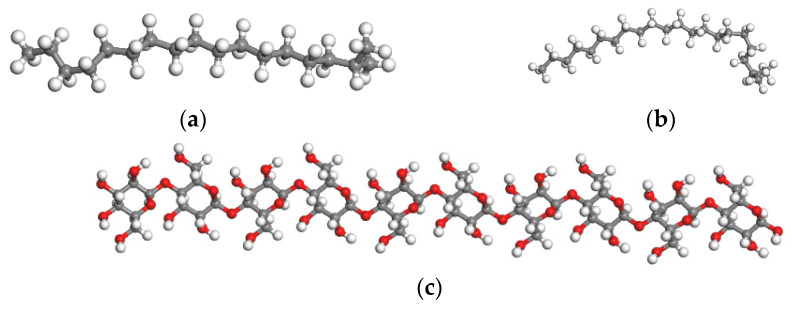
Molecular structures of BHOs and cellulose: (**a**) short-chain BHO; (**b**) long-chain BHO; (**c**) cellulose molecule.

The initial molecular structures of the short-chain BHO, long-chain BHO, and cellulose were constructed using the Amorphous Cell module in Materials Studio, and the molecular structures are shown in Figure 4. In the molecular models, gray, white, and red spheres represent carbon, hydrogen, and oxygen atoms, respectively.

**Figure 5:**
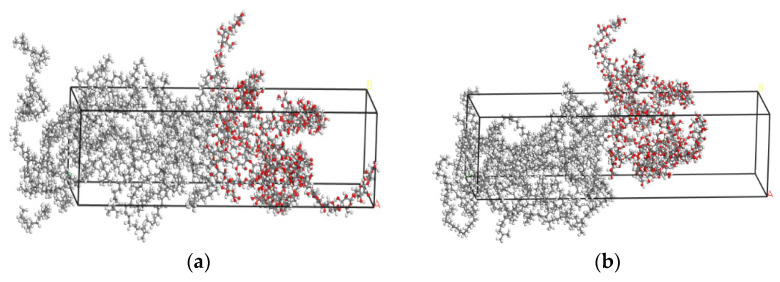
Oil-paper insulation composite model: (**a**) C18 BHO system; (**b**) C22 BHO system.

Subsequently, the oil-paper composite insulation systems were constructed based on the insulating oil and cellulose models, and the Build Layer tool was used to establish the oil-paper composite models. To obtain stable and reasonable initial configurations, geometric optimization, annealing treatment, and dynamic equilibration were first carried out using the Forcite module before the ReaxFF decomposition simulations, and all these procedures employed the COMPASS force field. Geometric optimization was performed using the Smart algorithm with a maximum of 10,000 iteration steps to ensure that the system energy reached a locally stable state. Subsequently, five cyclic annealing processes were conducted in the temperature range of 300 to 500 K under the NVT ensemble, and energy minimization was performed after each annealing cycle. During this process, van der Waals interactions were calculated using the Atom-based method, while electrostatic interactions were treated using the Ewald method. Finally, dynamic relaxation was carried out for 500 ps under the NPT ensemble at a pressure of 101.325 kPa, and the pressure was controlled using the Berendsen method. A stable oil-paper composite insulation model was thus obtained, as shown in Figure 5.

Reactive molecular dynamics simulations were performed on the LAMMPS platform. The simulations employed the NVT ensemble and periodic boundary conditions, and the CHO.ff reactive force field was selected. The simulation time step was set to 0.1 fs, and the temperature damping parameter was set to 10 fs. Compared with conventional nonreactive molecular dynamics, ReaxFF simulations require higher computational cost because bond order, charge distribution, and reaction information are updated dynamically during the simulation. In this study, the total simulation time was 500 ps, corresponding to 5.0 × 10^6^ molecular dynamics steps for each system. To ensure reproducibility and comparability, the two BHO systems were simulated using the same modeling procedure, equilibration process, reactive force field, time step, temperature, electric field strength, total simulation time, and species analysis criteria. It should also be noted that ReaxFF results may be sensitive to force field parameters, and different parameter sets may affect the exact reaction time, intermediate concentration, and absolute decomposition rate. Therefore, the simulation results were mainly used to compare relative degradation behaviors and identify possible reaction pathways rather than to determine absolute kinetic parameters. Because there is a significant time scale difference between molecular dynamics simulations and the actual operation process of transformers, it is difficult to directly observe obvious cellulose chain scission and small molecule generation within a limited simulation time under normal operating temperature and electric field conditions. Therefore, high temperature and a strong electric field were adopted as acceleration strategies to promote typical decomposition reactions and to compare the effects of different insulating oil molecular environments on cellulose decomposition pathways.

Before the formal simulations, preliminary ReaxFF simulations were first carried out at 1800 K and an electric field strength of 8 kV/mm. The results showed that under these relatively low stress conditions, neither oil-paper composite system exhibited obvious chemical bond cleavage or stable decomposition product formation. Subsequently, the simulation temperature and electric field strength were gradually increased. When the simulation conditions reached 2200 K and 350 kV/mm, stable chemical bond cleavage processes and representative decomposition products could be captured in both systems. In reactive molecular dynamics simulations, increasing the simulation temperature and electric field mainly affects the reaction rate and time scale, while the decomposition pathways and reaction mechanisms remain unchanged [28]. Therefore, the final simulation temperature and electric field strength were set to 2200 K and 350 kV/mm, respectively, and the total simulation time was 500 ps. These conditions were used to accelerate the electric field-induced degradation process and reveal the relative differences in cellulose decomposition pathways and product evolution behaviors under different BHO environments. It should be noted that although the applied temperature and electric field strength were much higher than those under actual operating conditions, this acceleration strategy has been widely validated in previous studies [29,30].

## 3. Results and Analysis

### 3.1. Partial Discharge Gas Generation Analysis

**Figure 6:**
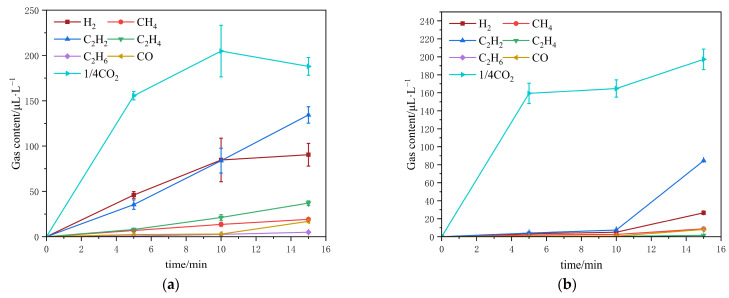
Evolution of characteristic gases under partial discharge in different BHO systems: (**a**) short-chain BHO system; (**b**) long-chain BHO system.

Figure 6 shows the evolution characteristics of gases generated from oil-paper composite insulation samples in short-chain and long-chain BHO systems during partial discharge. As the discharge time increased from 5 min to 15 min, the concentrations of H_2_, CH_4_, C_2_H_2_, C_2_H_4_, C_2_H_6_, CO, and CO_2_ in both systems generally increased. This result indicates that the high energy electrons, active radicals, and local thermal effects generated during partial discharge continuously induced the decomposition of insulating oil molecules and cellulose polymers. Under the strong nonuniform electric field at the needle plate defect, glycosidic bonds, C-O bonds, and C-H bonds in the cellulose backbone gradually cleaved, and various small molecular gases were subsequently generated through dehydrogenation, dehydration, decarbonylation, decarboxylation, and radical recombination reactions.

From the gas composition results, CO_2_ was the dominant gas product in both systems, and its concentration was significantly higher than those of the other characteristic gases, whereas C_2_H_6_ showed the lowest concentration. The high CO_2_ concentration indicates that oxygen-containing structures in cellulose polymers readily underwent decarboxylation and oxidative decomposition during partial discharge, making CO_2_ the major oxygen-containing gas generated in the oil-paper composite insulation system. In contrast, the low concentration of C_2_H_6_ suggests that the system preferentially generated H_2_, CO, CO_2_, and unsaturated hydrocarbon gases under partial discharge conditions, whereas the formation of saturated hydrocarbon products was relatively limited. Comparison of the two systems further shows that most characteristic gases, except CO_2_, exhibited significantly higher concentrations in the short-chain oil system than in the long-chain oil system, particularly H_2_, C_2_H_2_, C_2_H_4_, and CO. After 15 min of discharge, the H_2_ concentration in the short-chain oil system was approximately 3.4-times that in the long-chain oil system, while the CO concentration was approximately 2.1-times higher. These results indicate that dehydrogenation reactions, radical coupling processes, and decarbonylation reactions of oxygen-containing intermediates were more active in the short-chain oil system. This finding suggests that short-chain oil molecules more readily participated in C-H bond cleavage and hydrogen transfer processes during partial discharge, thereby providing more active hydrogen sources for the further reactions of cellulose decomposition fragments and promoting the formation of small molecular gases.

Among the hydrocarbon gases, the concentrations of unsaturated gases such as C_2_H_2_ and C_2_H_4_ increased rapidly with discharge time, and their concentrations in the short-chain oil system remained consistently higher than those in the long-chain oil system. The continuous increase in C_2_H_2_ and C_2_H_4_ indicates that stronger chain scission, dehydrogenation, and unsaturation reactions gradually occurred as partial discharge developed. In contrast, the generation amounts of CH_4_ and C_2_H_6_ were relatively low. This result suggests that although alkyl radicals could form saturated hydrocarbon gases through hydrogen abstraction reactions, decomposition intermediates under partial discharge conditions were more likely to undergo further dehydrogenation and form unsaturated small molecular products.

Compared with hydrocarbon gases, CO and CO_2_ more directly reflect the decomposition behavior of oxygen-containing structures in cellulose polymers. The short-chain oil system exhibited a significantly higher CO generation amount than the long-chain oil system, indicating that oxygen-containing decomposition intermediates in the short-chain oil environment underwent decarbonylation reactions to a greater extent. Unlike CO, the evolution trends of CO_2_ differed between the two systems. In the short-chain oil system, the CO_2_ concentration decreased after 10 min of discharge, whereas in the long-chain oil system, the CO_2_ concentration continuously increased with discharge time. This phenomenon indicates that in the later stage of discharge, some oxygen-containing intermediates in the short-chain oil system did not continue to oxidize into CO_2_, but were further converted into CO, H_2_, and unsaturated hydrocarbon small molecules. In contrast, oxygen-containing structures in the long-chain oil system were more likely to continuously undergo decarboxylation or oxidative decomposition and accumulate as CO_2_.

### 3.2. Cellulose Polymer Pyrolysis Pathway Analysis

**Figure 7:**
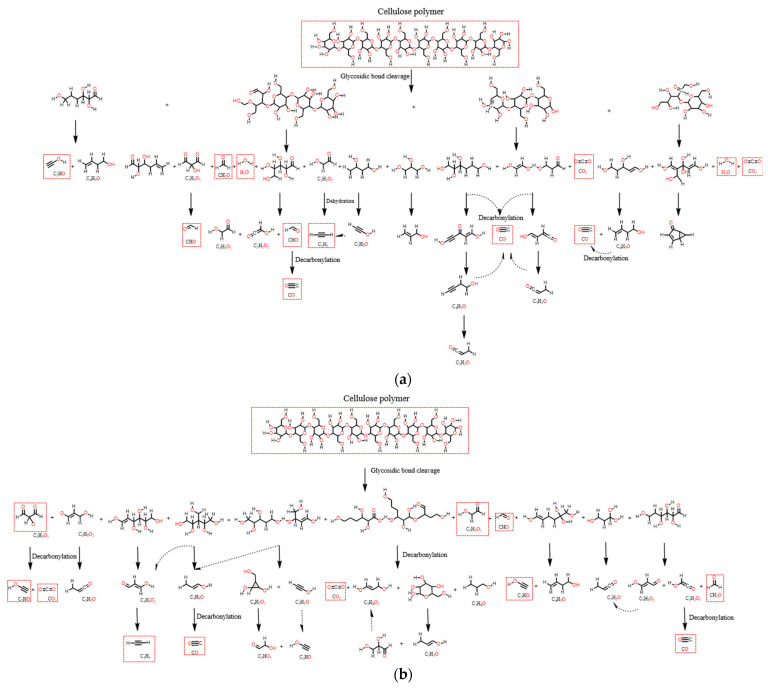
Main decomposition pathways of cellulose polymer in different BHO systems: (**a**) short-chain BHO system; (**b**) long-chain BHO system. Red atoms represent oxygen atoms, and red dashed boxes highlight key intermediates and characteristic gas products. Solid arrows indicate the main reaction pathways, while dashed arrows indicate simplified connections for convergent formation pathways leading to the same small-molecule product.

Figure 7 shows the main decomposition pathways of cellulose polymers in short-chain and long-chain BHO environments. The ReaxFF reactive molecular dynamics simulation results indicate that cellulose polymers in both systems first underwent glycosidic bond cleavage and oxygen-containing side group dissociation, followed by dehydration, decarbonylation, decarboxylation, chain scission, and radical recombination reactions, which further generated CO, CO_2_, H_2_O, C_2_H_2_, and various low carbon oxygen-containing intermediates. Although the fundamental decomposition reaction types in the two systems were similar, different oil molecular environments significantly affected the initial cellulose chain scission mode, intermediate distribution, and subsequent small molecule conversion process.

In the short-chain oil system, cellulose macromolecules first decomposed into relatively large oxygen-containing fragments, which were subsequently converted into low carbon oxygen-containing intermediates such as C_2_H_3_O_2_, CH_2_O, and CHO. Among these intermediates, CHO could generate CO through decarbonylation reactions, while fragments such as C_2_H_3_O_2_ could be further transformed into unsaturated small molecular products such as C_2_H_2_. In addition, some larger oxygen-containing fragments could further generate intermediates such as C_6_H_9_O_4_, C_5_H_4_O_3_, and C_4_H_3_O, and gradually release CO through continuous decarbonylation processes. This process indicates that cellulose decomposition in the short-chain oil system did not directly proceed to small molecule formation in a single step but instead exhibited a distinct sequential decomposition characteristic. Large oxygen-containing fragments were gradually converted into intermediate oxygen-containing fragments and were then further transformed into CO and unsaturated small molecular gas precursors. This phenomenon explains the higher generation amounts of CO, C_2_H_2_, and C_2_H_4_ observed in the short-chain oil system during the experiments, indicating that the short-chain oil environment more readily promoted the further conversion of cellulose decomposition intermediates into gaseous products.

Compared with the short-chain oil system, cellulose in the long-chain oil system more readily formed low carbon oxygen-containing intermediates and small molecular oxygen-containing fragments immediately after the initial chain scission process. After cellulose decomposition, low carbon oxygen-containing fragments such as C_3_H_5_O_2_, C_3_H_3_O_3_, C_2_H_3_O_2_, and CHO were generated at an earlier stage. Among them, C_3_H_3_O_3_ could generate CO_2_ through decarboxylation reactions, and this pathway corresponds to the continuous increase in CO_2_ observed experimentally in the long-chain oil system during discharge. Oxygen-containing fragments such as C_3_H_3_O and C_2_H_2_O_2_ could also further generate CO through decarbonylation reactions, but these pathways mainly occurred during the conversion of low carbon oxygen-containing fragments. This result indicates that the long-chain oil system more readily promoted the transformation of oxygen-containing cellulose structures into C_1_-C_5_ small molecular oxygen-containing fragments. Meanwhile, medium-sized fragments such as C_6_H_10_O_5_, C_6_H_11_O_5_, and C_9_H_15_O_7_ in the long-chain oil system could further decompose into low carbon oxygen-containing intermediates such as C_3_H_3_O_2_, C_4_H_3_O_3_, C_2_H_2_O, and C_2_HO, further demonstrating a stronger small molecule conversion tendency. Although these intermediates could continue to generate CO_2_ and CO, their conversion pathways toward gaseous products such as H_2_, C_2_H_2_, and C_2_H_4_ were less significant than those in the short-chain oil system. This result is consistent with the experimental observation that the long-chain oil system produced lower concentrations of most characteristic gases except CO_2_.

### 3.3. Analysis of Cellulose Pyrolysis Rate and Number of Oxygen-Containing Small Molecules

To quantitatively compare the decomposition degree of cellulose polymers in short-chain and long-chain BHO environments, statistical analyses of cellulose chains, oxygen-containing organic fragments, C_1_-C_5_ oxygen-containing small molecules, and the small molecule conversion ratio *R*_small_ were conducted based on ReaxFF reactive molecular dynamics simulations. Since BHOs are mainly composed of carbon and hydrogen elements, whereas cellulose polymers contain oxygen-containing functional groups, organic fragments containing C, H, and O elements were regarded as cellulose decomposition related products in this study. Although direct experimental measurements of cellulose degree of polymerization and mechanical property deterioration were not performed, these molecular level indicators can provide useful information on cellulose structural degradation. The number of cellulose chains was used to characterize the initial backbone cleavage process of cellulose. The number of oxygen-containing organic fragments was used to characterize the fragmentation degree of cellulose, while the number of C_1_-C_5_ oxygen-containing small molecules was used to evaluate the conversion degree of cellulose decomposition products toward low carbon small molecules.

To further characterize the relative conversion degree of cellulose decomposition products from intermediate oxygen containing fragments into low carbon small molecules, the small molecule conversion ratio *R*_small_ was defined as shown in Equation (1):

(1) $$R_{\mathrm{small}}=\frac{N_{\left(C1\text{-}C5,O\right)}}{N_{\left(\text{O-fragments}\right)}}$$

where *N*_(C1-C5,O)_ represents the number of C_1_-C_5_ oxygen-containing small molecules, and *N*_(O-fragments)_ represents the total number of oxygen-containing organic fragments. A higher ratio indicates that a larger proportion of low carbon small molecules exists among the oxygen-containing decomposition fragments, corresponding to a higher small molecule conversion degree of cellulose decomposition products. It should be noted that the decomposition rate discussed in this study represents an apparent decomposition rate obtained under accelerated ReaxFF simulation conditions involving high temperature and strong electric field stress. The purpose was to compare the relative differences in cellulose decomposition behavior between the two oil molecular environments rather than determine the absolute decomposition rate under actual operating conditions.

**Figure 8:**
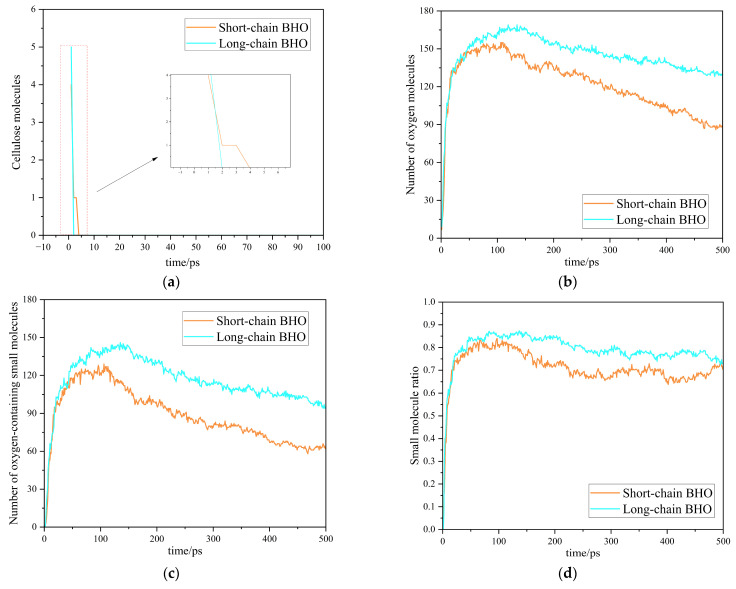
Evolution of cellulose degradation indicators in different BHO systems: (**a**) number of cellulose chains; (**b**) number of oxygen-containing organic fragments; (**c**) number of C_1_-C_5_ oxygen-containing small molecules; (**d**) small-molecule conversion ratio *R*_small_.

As shown in Figure 8a, cellulose chains in both systems rapidly decomposed during the initial simulation stage, indicating that the coupled high temperature and strong electric field conditions could rapidly induce glycosidic bond cleavage and C-O bond dissociation in the cellulose backbone. In comparison, cellulose chains in the long-chain oil system completely disappeared at 2 ps, whereas a small number of cellulose chains still remained in the short-chain oil system until 3 ps and completely decomposed after 4 ps. This result indicates that the initial cellulose chain scission process in the long-chain oil system was slightly faster. This phenomenon is consistent with the decomposition pathway analysis showing that the long-chain oil system more readily formed low carbon oxygen-containing intermediates directly, suggesting that local cleavage of oxygen containing cellulose structures proceeded more rapidly in the long-chain oil environment.

As shown in Figure 8b, the number of oxygen-containing organic fragments in both systems rapidly increased during the initial reaction stage, indicating that a large number of oxygen-containing decomposition fragments were quickly generated after cellulose backbone cleavage. Compared with the short-chain oil system, the long-chain oil system exhibited a generally higher number of oxygen-containing organic fragments. Its maximum value reached approximately 169, which was higher than the value of 155 observed in the short-chain oil system. At 500 ps, the number of oxygen-containing organic fragments in the long-chain oil system still remained at 129, whereas the value in the short-chain oil system decreased to 89. This result further suggests that the long-chain oil environment more readily promoted continuous fragmentation of oxygen-containing cellulose structures, which is consistent with the pathway analysis showing earlier formation of low carbon oxygen-containing intermediates and a more pronounced small molecule conversion tendency in the long-chain oil system.

As shown in Figure 8c, the number of C_1_-C_5_ oxygen-containing small molecules in the long-chain oil system remained higher than that in the short-chain oil system during most of the simulation period. The maximum value in the long-chain oil system reached approximately 146, which was higher than the value of 129 observed in the short-chain oil system. At 500 ps, the number of C_1_-C_5_ oxygen-containing small molecules in the long-chain oil system was still 94, whereas the corresponding value in the short-chain oil system was 63. These results indicate that oxygen-containing intermediates generated from cellulose decomposition in the long-chain oil system more readily underwent further chain scission, dehydration, decarbonylation, and decarboxylation reactions, thereby converting into low-carbon oxygen-containing small molecules. This result is consistent with the continuously increasing CO_2_ observed experimentally in the long-chain oil system, suggesting that oxygen-containing decomposition products in the long-chain oil environment were more likely to accumulate as CO_2_ through decarboxylation and oxidative decomposition pathways rather than being extensively converted into gaseous products such as H_2_, C_2_H_2_, and C_2_H_4_.

Further comparison of the small molecule conversion ratio *R*_small_ shows that the long-chain oil system exhibited a higher small molecule conversion ratio during most reaction stages, as shown in Figure 8d. The maximum *R*_small_ value in the short-chain oil system was approximately 0.840, whereas the maximum value in the long-chain oil system reached approximately 0.873. Meanwhile, at typical simulation times of 50, 100, 200, 300, and 400 ps, the *R*_small_ values in the long-chain oil system were consistently higher than those in the short-chain oil system. These results indicate that the long-chain oil system not only generated a larger number of oxygen-containing fragments but also allowed a greater proportion of these fragments to be converted into C_1_-C_5_ low-carbon oxygen-containing small molecules. In contrast, although the short-chain oil system exhibited a relatively lower small molecule conversion ratio, it generated higher amounts of H_2_, CO, C_2_H_2_, and C_2_H_4_ during the partial discharge experiments. This result suggests that the short-chain oil environment more readily promoted further dehydrogenation, decarbonylation, and unsaturation reactions of decomposition intermediates, thereby facilitating their conversion into gaseous products.

### 3.4. Analysis of the Generation Pathways of Characteristic Gases in Cellulose

To further reveal the formation mechanisms of characteristic gases from cellulose polymers under electrical and thermal stress, the microscopic formation pathways of CO_2_, CO, H_2_, CH_4_, C_2_H_2_, C_2_H_4_, and C_2_H_6_ from cellulose were analyzed based on ReaxFF reactive molecular dynamics simulations.

**Figure 9:**
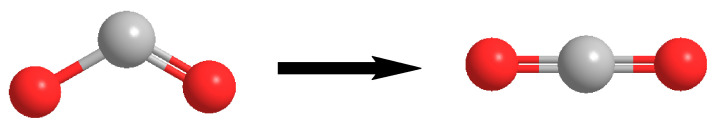
Formation pathway of CO_2_ during cellulose decomposition.

Figure 9 shows the formation pathway of CO_2_. The CO_2_ formation pathways in the two oil environments were basically consistent. In both systems, oxygen-containing fragments generated from cellulose decomposition underwent C-O bond rearrangement and formed stable linear CO_2_ molecules. This process essentially corresponds to decarboxylation or oxidative decomposition of oxygen containing functional groups in cellulose, indicating that CO_2_ mainly originated from the cleavage of oxygen containing structures such as carboxyl, carbonyl, and ether groups in cellulose polymers. This pathway is consistent with the high CO_2_ concentrations observed in both systems during the partial discharge experiments.

**Figure 10:**
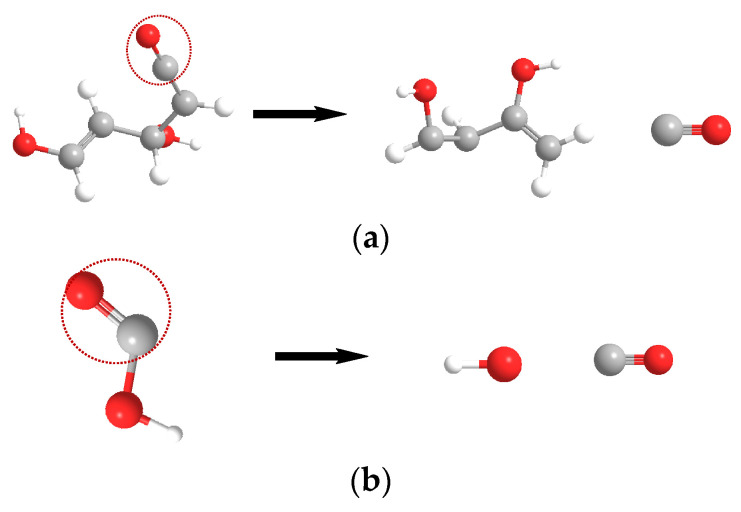
Formation pathways of CO during cellulose decomposition in different BHO systems: (**a**) short-chain BHO system; (**b**) long-chain BHO system.

Figure 10 shows the formation pathway of CO. In the short-chain oil environment, CO was mainly generated through continuous decarbonylation of relatively large oxygen-containing chain intermediates. After cellulose decomposition, chain intermediates containing hydroxyl and carbonyl structures further underwent C-C bond cleavage, causing the terminal carbonyl group to detach in the form of CO. In contrast, the CO formation pathway in the long-chain oil environment was more direct. CO was mainly generated from small molecular formyl or carboxyl type intermediates through C-O bond cleavage, accompanied by the formation of hydroxyl radicals. This difference indicates that oxygen-containing cellulose intermediates in the short-chain oil environment were more likely to undergo multistep decomposition and continuous decarbonylation, which agrees with the higher CO concentration observed in the short-chain oil system than in the long-chain oil system.

**Figure 11:**
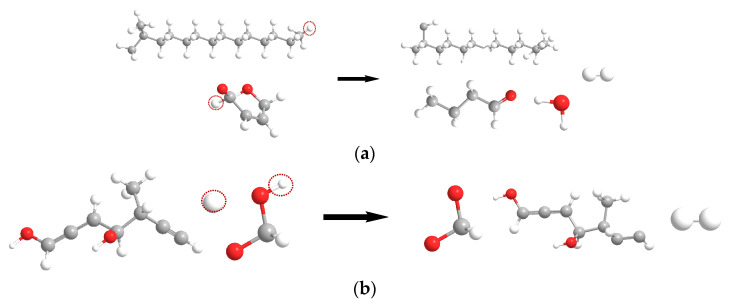
Formation pathways of H_2_ during cellulose decomposition in different BHO systems: (**a**) short-chain BHO system; (**b**) long-chain BHO system.

Figure 11 shows the formation pathway of H_2_. In the short-chain oil environment, oxygen-containing fragments generated from cellulose decomposition could undergo dehydration, decarbonylation, and structural rearrangement, accompanied by the release of active hydrogen radicals. Subsequently, two hydrogen radicals coupled to form H_2_. In the long-chain oil environment, H_2_ mainly originated from hydrogen transfer reactions within cellulose decomposition fragments. For example, unsaturated oxygen-containing fragments or formic acid-based intermediates could react with free hydrogen radicals to generate H_2_. By comparison, cellulose decomposition fragments in the short-chain oil environment were more prone to dehydrogenation and radical coupling processes, which corresponds to the significantly higher H_2_ concentration observed in the short-chain oil system during the experiments.

**Figure 12:**
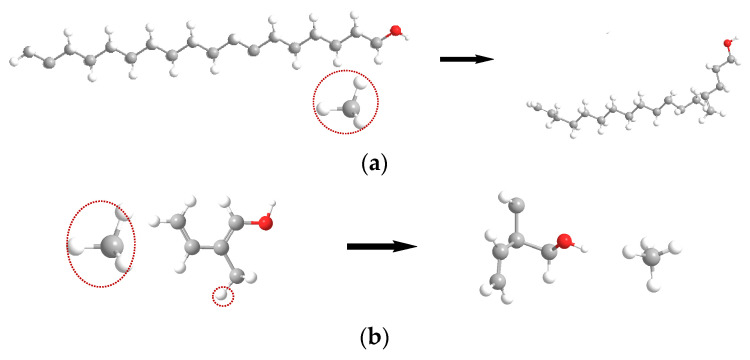
Formation pathways of CH_4_ during cellulose decomposition in different BHO systems: (**a**) short-chain BHO system; (**b**) long-chain BHO system.

Figure 12 shows the formation pathway of CH_4_. In both oil environments, CH_4_ formation was related to methyl radicals generated during cellulose decomposition. In the short-chain oil environment, methyl radicals were more likely to form CH_4_ through hydrogen abstraction reactions, accompanied by the unsaturation transformation of oxygen containing chain fragments. In the long-chain oil environment, CH_4_ was mainly formed by hydrogen abstraction of methyl radicals from low carbon oxygen-containing cellulose fragments. Since cellulose decomposition products in the long-chain oil environment tended to form low-carbon oxygen-containing fragments, while the pathways for hydrogen abstraction and saturation of methyl radicals were relatively limited, the CH_4_ generation amount in the long-chain oil system was lower than that in the short-chain oil system.

**Figure 13:**
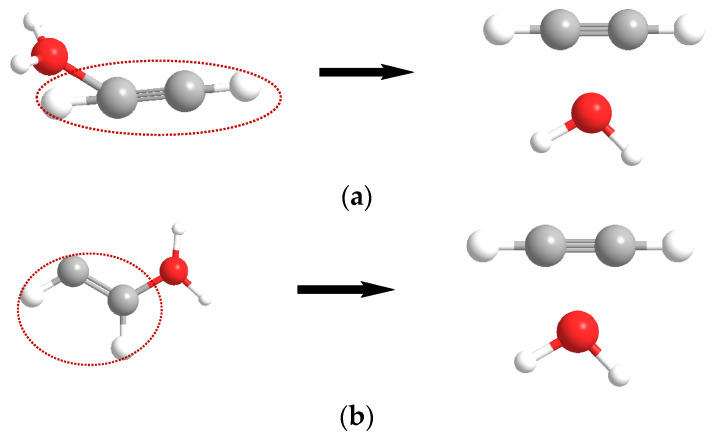
Formation pathways of C_2_H_2_ during cellulose decomposition in different BHO systems: (**a**) short-chain BHO system; (**b**) long-chain BHO system.

Figure 13 shows the formation pathway of C_2_H_2_. In the short-chain oil environment, C_2_H_2_ was mainly generated from alkynol-type oxygen containing intermediates produced by cellulose decomposition through dehydration reactions. In this process, hydroxyl groups and adjacent hydrogen atoms were removed to form H_2_O, while the C≡C structure was retained to generate C_2_H_2_. In the long-chain oil environment, C_2_H_2_ was mainly generated from enol-type intermediates through further dehydration and dehydrogenation. The precursor in the long-chain oil system had a lower degree of unsaturation than that in the short-chain oil system and required further structural rearrangement to form the alkyne bond. This pathway difference indicates that cellulose decomposition fragments in the short-chain oil environment had a stronger tendency toward unsaturation, which is consistent with the higher C_2_H_2_ generation amounts observed in the short-chain oil system during the experiments.

**Figure 14:**
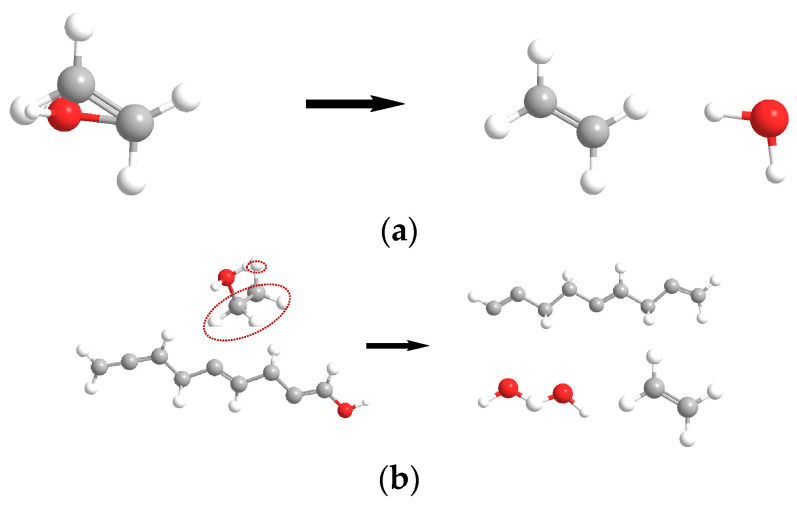
Formation pathways of C_2_H_4_ during cellulose decomposition in different BHO systems: (**a**) short-chain BHO system; (**b**) long-chain BHO system.

Figure 14 shows the formation pathway of C_2_H_4_. In the short-chain oil environment, C_2_H_4_ could be directly generated from vinyl alcohol type intermediates produced by cellulose decomposition through dehydration. In the long-chain oil environment, C_2_H_4_ formation mainly involved further dehydration and structural rearrangement of ethanol type fragments generated from cellulose decomposition. Compared with the short-chain oil environment, C_2_H_4_ formation in the long-chain oil environment depended more strongly on rearrangement of intermediates, resulting in relatively lower formation efficiency. This behavior agrees with the experimental observation that the C_2_H_4_ concentration in the short-chain oil system was higher than that in the long-chain oil system.

**Figure 15:**
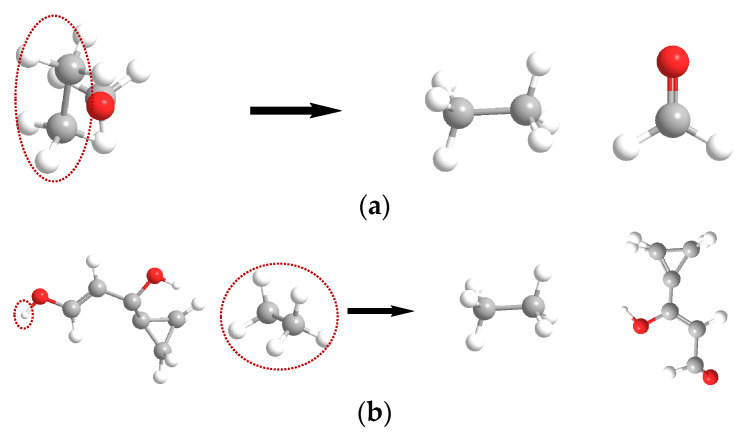
Formation pathways of C_2_H_6_ during cellulose decomposition in different BHO systems: (**a**) short-chain BHO system; (**b**) long-chain BHO system.

Figure 15 shows the formation pathway of C_2_H_6_. In the short-chain oil environment, C_2_H_6_ could be directly generated from methoxy or alkyl structure fragments produced by cellulose decomposition through β scission, accompanied by the formation of formaldehyde-type oxygen-containing byproducts. In the long-chain oil environment, C_2_H_6_ was mainly generated through hydrogen abstraction by ethyl radicals from cyclic oxygen containing unsaturated fragments, accompanied by rearrangement of oxygen-containing cyclic structures. Since C_2_H_6_ formation in the long-chain oil environment depended on radical hydrogen abstraction and structural rearrangement, its pathway was relatively complex and its generation amount was lower. This result is consistent with the experimental phenomenon that C_2_H_6_ concentrations were low in both systems and were lower in the long-chain oil system than in the short-chain oil system.

The proposed gas generation pathways were inferred from ReaxFF simulation trajectories, but their validity was further supported by the experimental gas evolution results and previous studies on cellulose decomposition. Experimentally, CO_2_ was the dominant gas product in both BHO systems, which is consistent with the simulated pathway in which CO_2_ mainly originates from decarboxylation or oxidative decomposition of oxygen-containing cellulose fragments. The higher concentrations of H_2_, CO, C_2_H_2_, and C_2_H_4_ in the short-chain BHO system also agree with the simulated stronger hydrogen transfer, continuous decarbonylation, and unsaturation reactions. In contrast, C_2_H_6_ showed the lowest concentration in both systems, corresponding to the limited saturated hydrocarbon formation through radical recombination under partial discharge conditions. Previous studies have shown that CO and CO_2_ are mainly associated with the cleavage and decomposition of oxygen-containing structures in cellulose, while H_2_ and low molecular weight hydrocarbon gases are related to dehydrogenation, chain scission, and radical reactions [14,28]. Therefore, although the gas generation pathways were derived from accelerated ReaxFF simulations, their consistency with both experimental gas trends and established cellulose decomposition mechanisms supports their reliability as reasonable molecular level explanations.

## 4. Discussion

The present study demonstrates that BHO molecules should not be regarded merely as inert surrounding media in the oil-paper composite insulation system. Instead, they affect cellulose decomposition by regulating the local reaction environment, radical transfer behavior, hydrogen transfer, and subsequent conversion of cellulose-derived intermediates. In addition to molecular chain structure, the physicochemical properties of the two BHOs may also influence gas generation behavior. The kinematic viscosity of the short-chain BHO was 3.68 mm^2^/s at 40 °C, whereas that of the long-chain BHO was 9.93 mm^2^/s. Thus, the viscosity of the long-chain BHO was approximately 2.70-times that of the short-chain BHO. This large difference suggests that the lower viscosity of the short-chain BHO may facilitate molecular diffusion, radical contact, and hydrogen transfer between oil molecules and cellulose-derived intermediates, thereby promoting the formation of H_2_, CO, C_2_H_2_, and C_2_H_4_. By contrast, the dielectric constants of the short-chain and long-chain BHOs were 1.91 and 1.95, respectively, corresponding to a difference of only about 2.1%. Their moisture contents were 16.7 ppm and 18.4 ppm, respectively, with the long-chain BHO being about 10.2% higher. Compared with the viscosity difference, the differences in dielectric constant and moisture content were relatively small. Therefore, although the dielectric constant and moisture content may affect partial discharge behavior and radical reactions to some extent, the gas generation differences observed in this study are more likely related to the combined effects of BHO chain structure, viscosity-controlled molecular mobility, and cellulose-derived intermediate evolution.

In the short-chain BHO system, the lower kinematic viscosity may facilitate molecular diffusion, radical contact, and hydrogen transfer between oil molecules and cellulose-derived radicals. Hydrogen species released from C-H bond cleavage of short-chain BHO molecules can participate in hydrogen abstraction and radical recombination reactions, thereby promoting H_2_ and CH_4_ formation. Meanwhile, the enhanced radical activity in this system promotes continuous decarbonylation and unsaturation reactions of oxygen-containing cellulose fragments, leading to higher CO, C_2_H_2_, and C_2_H_4_ generation. By contrast, the long-chain BHO system promotes a different decomposition route. The ReaxFF results show that cellulose chains disappear slightly faster, and more oxygen-containing organic fragments and C_1_-C_5_ oxygenated molecules are formed in the long-chain BHO environment. This indicates that the long-chain BHO favors the fragmentation and low-carbon conversion of cellulose oxygen-containing structures. However, these fragments are mainly converted into low-carbon oxygenated intermediates rather than gaseous products. The higher viscosity of the long-chain BHO may further limit molecular diffusion, radical contact, and hydrogen transfer between oil molecules and cellulose-derived intermediates, thereby reducing the further conversion of oxygen-containing intermediates into H_2_, C_2_H_2_, C_2_H_4_, and other hydrocarbon gases. Therefore, the long-chain BHO promotes greater formation of oxygen-containing fragments while generating lower concentrations of most characteristic gases except CO_2_. This result suggests that the long-chain BHO does not simply suppress cellulose degradation but changes the dominant conversion route of cellulose-derived intermediates.

It should be noted that a longer BHO molecule does not necessarily indicate better insulation performance or weaker cellulose degradation. The results of this study show that the short-chain and long-chain BHOs each have different effects on cellulose degradation and gas generation behavior. The short-chain BHO has lower viscosity, which may be beneficial for molecular mobility and heat transfer, but it also favors hydrogen transfer, decarbonylation, and unsaturation reactions, leading to higher H_2_, CO, C_2_H_2_, and C_2_H_4_ generation. The long-chain BHO can reduce the generation of most characteristic gases except CO_2_, but it favors cellulose fragmentation and low-carbon oxygenated intermediate formation. Therefore, the optimal BHO chain length should be determined by the comprehensive balance among dielectric performance, viscosity, thermal stability, heat transfer ability, gas generation tendency, and its influence on cellulose degradation pathways.

From a practical perspective, these findings provide useful implications for transformer insulation diagnostics, condition monitoring, and BHO selection. The results suggest that the molecular environment of BHO can affect the gas generation fingerprint of oil-paper insulation under partial discharge. In the short-chain BHO system, the higher generation of H_2_, CO, C_2_H_2_, and C_2_H_4_ indicates that these gases may serve as sensitive indicators of partial-discharge-induced cellulose degradation in this oil environment. In contrast, the lower concentrations of most characteristic gases except CO_2_ in the long-chain BHO system should not be simply interpreted as weaker cellulose degradation, because the ReaxFF results indicate greater formation of oxygen-containing fragments and C_1_-C_5_ oxygenated molecules. Therefore, condition monitoring of BHO-based oil-paper insulation systems should consider the influence of oil molecular structure on cellulose decomposition pathways, rather than relying only on gas concentration levels. For insulating oil selection, BHO chain length should be evaluated by balancing dielectric performance, viscosity, thermal stability, heat transfer ability, gas generation tendency, and its influence on cellulose degradation pathways. These findings may provide molecular-level support for fault diagnosis and molecular structure optimization of environmentally friendly BHO-based transformer insulating oils.

Several limitations should also be noted. Intermediate chain lengths may exhibit transitional behavior between the short-chain and long-chain BHO systems, such as intermediate viscosity, gas generation tendency, and cellulose-derived intermediate evolution. Further studies involving more BHO samples with intermediate chain lengths are required to establish a more complete relationship between BHO chain length, insulation performance, and cellulose degradation behavior. It should be noted that this short-term experiment represents an accelerated severe-defect condition rather than long-term transformer operation. The results should not be directly used to predict the service lifetime of transformer insulation. Nevertheless, the clear differences in H_2_, CO, C_2_H_2_, and C_2_H_4_ generation within this period indicate that the BHO molecular environment can influence the initial degradation response of cellulose under partial discharge. Since partial discharge is an important precursor of insulation deterioration, these short-term gas evolution characteristics can provide useful information for early-stage condition monitoring and comparative evaluation of insulating oil systems.

Although the ReaxFF simulations were conducted under accelerated temperature and electric field conditions, the results were used mainly to compare relative degradation pathways rather than to predict the actual degradation rate under service conditions. The consistency between the simulated reaction pathways and the experimental gas evolution trends supports their reliability as molecular level explanations for the different degradation behaviors in the two BHO systems. Future work will further combine dissolved gas analysis with experimental degree of polymerization measurement and mechanical property testing to establish a more complete relationship between gas generation behavior and cellulose structural degradation.

## 5. Conclusions

The main conclusions are summarized as follows:

The partial discharge experimental results showed that both oil-paper composite systems generated seven characteristic gases, including H_2_, CH_4_, C_2_H_2_, C_2_H_4_, C_2_H_6_, CO, and CO_2_. Among them, CO_2_ was the dominant gas product, while C_2_H_6_ exhibited the lowest concentration. The generation amounts of H_2_, CO, C_2_H_2_, and C_2_H_4_ in the short-chain oil system were significantly higher than those in the long-chain oil system. After 15 min of discharge, the H_2_ concentration in the short-chain oil system was approximately 3.4-times that in the long-chain oil system, and the CO concentration was approximately 2.1-times higher. These results indicate that the short-chain oil system exhibited stronger dehydrogenation, decarbonylation, and unsaturation reaction characteristics, which promoted the accumulation of small molecular gas products.

The ReaxFF simulation results suggested different cellulose decomposition behaviors in the two oil environments. The short-chain oil system exhibited more obvious stepwise chain scission and continuous decarbonylation, which promoted the formation of CO and unsaturated gas precursors. In contrast, the long-chain oil system tended to promote cellulose fragmentation, producing more oxygen-containing organic fragments and C_1_-C_5_ oxygen-containing small molecules, with a higher *R*_small_. These results indicate that the long-chain oil environment may favor oxygen-containing fragmentation and low-carbon small-molecule conversion rather than direct conversion into hydrocarbon gases.

Characteristic gas pathway analysis suggested that the seven gases could be generated from cellulose-derived intermediates through decarboxylation, decarbonylation, dehydrogenation, hydrogen abstraction, dehydration, and structural rearrangement. Overall, the short-chain oil environment was more likely to favor further gasification of cellulose decomposition intermediates, whereas the long-chain oil environment tended to favor oxygen-containing fragmentation and low-carbon small-molecule conversion. It should be noted that these molecular mechanisms were mainly inferred from accelerated ReaxFF simulations and supported by the experimental gas evolution trends. Therefore, they should be regarded as possible molecular-level degradation pathways and relative differences between the two BHO systems, rather than direct observations of cellulose degradation under practical transformer operating conditions.

## Figures and Tables

**Table 1:** Key properties of BHOs.

Parameter	Short-Chain BHO	Long-Chain BHO
Density (20 °C, g/cm^3^)	0.8	0.81
Moisture Content (ppm)	16.7	18.4
Kinematic Viscosity (40 °C, mm^2^/s)	3.68	9.93
Acid Value (mg KOH/g)	0.01	0.01
Pour Point (°C)	−25	−45
Flash Point (°C)	137	187
tan δ (90 °C)	0.00047	0.00022
Dielectric Constant (90 °C)	1.91	1.95

## Data Availability

Data are contained within the article.
